# Bridging Tumorigenesis and Therapy Resistance With a Non-Darwinian and Non-Lamarckian Mechanism of Adaptive Evolution

**DOI:** 10.3389/fonc.2021.732081

**Published:** 2021-09-10

**Authors:** Francesco Catania, Beata Ujvari, Benjamin Roche, Jean-Pascal Capp, Frédéric Thomas

**Affiliations:** ^1^Institute for Evolution and Biodiversity, University of Münster, Münster, Germany; ^2^Centre for Integrative Ecology, School of Life and Environmental Sciences, Deakin University, Deakin, VIC, Australia; ^3^CREEC/CANECEV, MIVEGEC (CREES), Centre de Recherches Ecologiques et Evolutives sur le Cancer, University of Montpellier, CNRS, IRD, Montpellier, France; ^4^Toulouse Biotechnology Institute, University of Toulouse, INSA, CNRS, INRAE, Toulouse, France

**Keywords:** tumor evolution, adaptation, cell growth, stress response, natural selection, environment, cancer therapy

## Abstract

Although neo-Darwinian (and less often Lamarckian) dynamics are regularly invoked to interpret cancer’s multifarious molecular profiles, they shine little light on how tumorigenesis unfolds and often fail to fully capture the frequency and breadth of resistance mechanisms. This uncertainty frames one of the most problematic gaps between science and practice in modern times. Here, we offer a theory of adaptive cancer evolution, which builds on a molecular mechanism that lies outside neo-Darwinian and Lamarckian schemes. This mechanism coherently integrates non-genetic and genetic changes, ecological and evolutionary time scales, and shifts the spotlight away from positive selection towards purifying selection, genetic drift, and the creative-disruptive power of environmental change. The surprisingly simple *use-it* or *lose-it* rationale of the proposed theory can help predict molecular dynamics during tumorigenesis. It also provides simple rules of thumb that should help improve therapeutic approaches in cancer.

## Introduction

Cancer cells are often thought of as a pile of aberrant genetic variants, hence the prevailing view of cancer as a genetic disease (1). However, cancer is also — and first of all — an adaptation to its microenvironment with a strong non-genetic component (2–10). Transcriptional plasticity and epigenetic heterogeneity are increasingly recognized as major players in the ability of cancer cells to evade therapies (11–19). This growing body of evidence revises the view of mutations as sole or principal drivers of drug resistance (20–26), and offers more than an additional perspective on an old problem. It implies that interpreting therapeutic resistance as an unambiguous indicator of selection of random beneficial mutations may be inaccurate. This potential inaccuracy may have direct and serious consequences for cancer patients. Moreover, as it is possible that the adaptive dynamics that foster therapeutic resistance also unfold during tumorigenesis, it calls for a more cautious interpretation of previous findings related to cancer initiation and progression.

## The Problem

Although non-genetic resistance to chemotherapy/target-selective drugs appears to be pervasive in cancer cells, the molecular mechanisms that drive this resistance are yet incompletely understood (12, 27). Non-genetic resistance can be acquired by environmental induction, *i.e.*, drug-induced epigenetic reconfigurations can enhance cells’ survival to the very therapeutic environment (28, 29). Alternatively, epigenetic configurations that are advantageous in the therapeutic environment may be pre-existing and undergo positive selection (30, 31). Moreover, while resistance can be coupled with purely epigenetic or purely genetic changes, it is also possible that initially non-genetic changes become genetically hardwired/assimilated over time, *i.e.*, an environmentally induced phenotype is made constitutive (12, 32, 33). In sum, cancer populations can achieve drug resistance through genetic and/or non-genetic means and *via* a mix of neo-Darwinian and *quasi*-Lamarckian mechanisms of adaptive evolution (a fully Lamarckian scheme of adaptation would entail, *inter alia*, that environmental factors cause directed adaptive changes).

This wealth of possible paths to resistance is confusing and consequently not good news for the design of effective cancer therapies. On the positive side, several observations (27, 34, 35) indicate that mechanisms similar to those that drive therapeutic resistance in cancer cells may also drive adaptive phases during the poorly understood process of tumorigenesis. Thus, the insights gained in the context of therapeutic resistance may shine light on tumorigenesis and help reveal some general principles and/or deterministic dynamics (36), which can in turn contribute to the development of more effective cancer prevention. Here, we explore this possibility. More specifically, we ask two questions: Are there distinct intracellular mechanisms and simple evolutionary dynamics (not necessarily neo-Darwinian or Lamarckian), which can help explain the progression of events during oncogenic transformation? And if so, can these mechanisms/dynamics have clinical utility and general implications for cancer evolution and therapy? An empirically supported positive answer to these questions can be given when the dominant and deep-rooted neo-Darwinian (ND) model of adaptive evolution is bypassed.

## A Short Overview of the Neo-Darwinian Model

The ND model is nearly universally invoked to explain how living beings adapt to their surroundings. In this model, which integrates Darwinian dynamics and Mendelian genetics, natural selection acts on spontaneous genetic mutations, favoring the spread of heritable variants that are advantageous in the prevailing conditions (37). Selectable genetic changes predate adaptation and chance plays a central role in the occurrence of genetic changes. The ND model is powerful: its implications are entrenched in modern thinking and far-reaching.

Against this background, it may come as no surprise that the ND model is also widely leveraged for explaining the genesis and the evolution of adaptive traits in cancer (21, 38–44). For example, positive selection is widely thought to promote recurring inactivating mutations in *TP53*, the most mutated gene across human cancer types (45). More generally, it is most often undisputed, let alone plausible, that accidental genomic variants that are advantageous in prevailing conditions drive adaptations of cancer cells to the tumor microenvironment and therapy resistance. The ND model serves as an evolutionary framework for cancer genomics studies to reconstruct clonal evolution: mutation and selection of new mutations that happen to be beneficial in the tumor microenvironment drive the expansion of subclones (46).

In the ND model, the speed of adaptation partly relies on the rate at which beneficial mutations appear, survive, and spread (47). As plausible as the fixation of beneficial mutations is, the chance of it happening may be miniscule (48). Beneficial (driver) mutations in cancer cells emerge and/or segregate in a context where most co-occurring (passenger) mutations are deleterious (44). Ubiquitous purifying selection is expected to most often purge these cells alongside possible beneficial mutations that frequently confer only a minimal selective advantage (49). Even when purifying selection is relaxed, the mere interaction of accruing deleterious mutations may facilitate adaptation (50, 51). Finally, random genetic drift can lead to the fixation of deleterious mutations by sheer chance (52–56), and this drift also limits the ability of selection to refine a phenotype (57). In short, it is certainly not a given that beneficial mutations, if present, spread and reach fixation. Consistent with this, no beneficial mutations followed by selective sweeps and clonal expansion were detected in recent cancer genomic studies (58, 59). Besides, when driver genes are reported [*e.g.,* (60)], the evidence that these typically highly mutated genes are positively selected for (often inactivating) tumorigenesis-driving mutations does not rule out alternative explanations (see below and **Box 1**). More generally, although selection of beneficial mutations has for long time provided the primary mechanistic account for the origin of adaptive phenotypic traits (61, 62), unambiguous cases of evolved adaptation driven by positive selection of both new rare beneficial mutations and standing genetic variation appear to be relatively rare (63) or unsubstantiated (64). In the majority of cases they rely largely or entirely on the statistical analysis of sequence data without a biological mechanism that underlay the presumed selection (65).

Box 1 **Is dN/dS > 1 irrefutable evidence for positive selection?**

The *use-it* or *lose-it* model does not exclude that positive selection may play a role in evolutionary adaptation. At the same time, it offers a null hypothesis against which the explanatory power of the neo-Darwinian framework can be measured. Genes with cancer-associated mutations in healthy tissues are a valuable test bed for assessing the explanatory power of the *use-it* or *lose-it* model *vs*. the commonly assumed neo-Darwinian dynamics.

In healthy somatic tissues, genes that operate as cancer drivers have been reported to preferentially accrue mutations and to be under positive selection (dN/dS > 1) (66). If dN/dS > 1 is irrefutable evidence for positive selection, then this finding is in contradiction with the *use-it* or *lose-it* model, so we decided to reexamine it. In the *use-it* or *lose-it* model, the mutational enrichment of cancer driver genes in healthy somatic tissues suggests that healthy cells and cancer cells can experience similar microenvironmental conditions, as expected given that cancer cells originate from healthy cells. It also implies that cancer-inducing microenvironmental conditions can occur throughout the human body without necessarily giving rise to cancer but presumably increasing the risk of developing it (67). Finally, in the *use-it* or *lose-it* model the preferential accumulation of cancer mutations in healthy tissues flags relaxed purifying selection (rather than positive selection) in the presence of a cancer-inducing environment. It predicts that the somatic genes under focus should accumulate inactivating mutations and exhibit dN/dS values ≈ 1.

Interestingly, genes with cancer-associated mutations in healthy tissues do exhibit dN/dS values ≈ 1 when missense mutations are examined. dN/dS values > 1 are only detected when inactivating mutations are considered (66). Thus, the putative signature of positive selection reported for cancer driver genes in healthy somatic tissues rests only on the preferential accrual of inactivating mutations, which is in line with the *use-it* or *lose-it* model. How about the dN/dS ratio >1, which is interpreted as indicating the presence of beneficial mutations? According to the *use-it* or *lose-it* model, this interpretation may be inaccurate. In addition to previously reported problems with using the dN/dS metric as an unambiguous indicator of evolutionary adaptation (68–70), a couple of remarks support this possibility. First, a focus on nonsense mutations can lead to an increased dN/dS ratio because termination codons are A + T rich, and in the human genome (as well as others) a mutational bias toward A + T has been found (71). Second, it is not clear how healthy cells, which lack a “self-defined” fitness (72), may benefit from selectively advantageous mutations. Back to the *use-it* or *lose-it* model, the inactivating mutations that accrue in cancer driver genes in healthy tissues are predicted to be effectively neutral because they *follow* the environmentally induced and epigenetically controlled manifestation of the adaptive (alternative) phenotype in the pre-tumor environment.

This raises a question that deserves careful consideration: does the ND model offer the only possible account for the evolution of adaptive traits? Shifting our focus on cancer, are there other equally viable mechanisms of adaptation, which could help explain how tumors emerge or therapy resistance is acquired (see also **Box 1**)? In a time where cancer is among the leading causes of death globally, an alternative or additional mechanism of adaptive evolution could offer new perspectives on how to interpret cancer genomic data and address cancer as a health problem. An additional and equally viable mechanism of adaptive evolution would also provide a null hypothesis against which the explanatory power of the ND model can be measured [reminiscent of the neutral theory of molecular evolution (73) and the mutational hazard hypothesis (74, 75)].

## A Mechanism of Adaptive Evolution Without Positive Selection

Like the ND model, a non-Darwinian model that describes how evolved adaptations may arise during tumorigenesis or in response to therapy should help explain and interpret a wide range of observations. Ideally, it should have several properties. For example, it should help integrate ubiquitous and well-established phenomena such as pleiotropy, plasticity, and trade-offs (76). Also, it should be explicit about the relative contribution of evolutionary forces in the onset of adaptations as well as the interaction between these forces and the environment [*e.g.*, surrounding tumor cells (77)]. Further, it should be able to integrate genetics and epigenetics, both of which play a central role in the emergence of cancer and cancer drug resistance [*e.g.,* (13)]. It should also generate testable hypotheses. Last, it should help make predictions.

**Figure 1:**
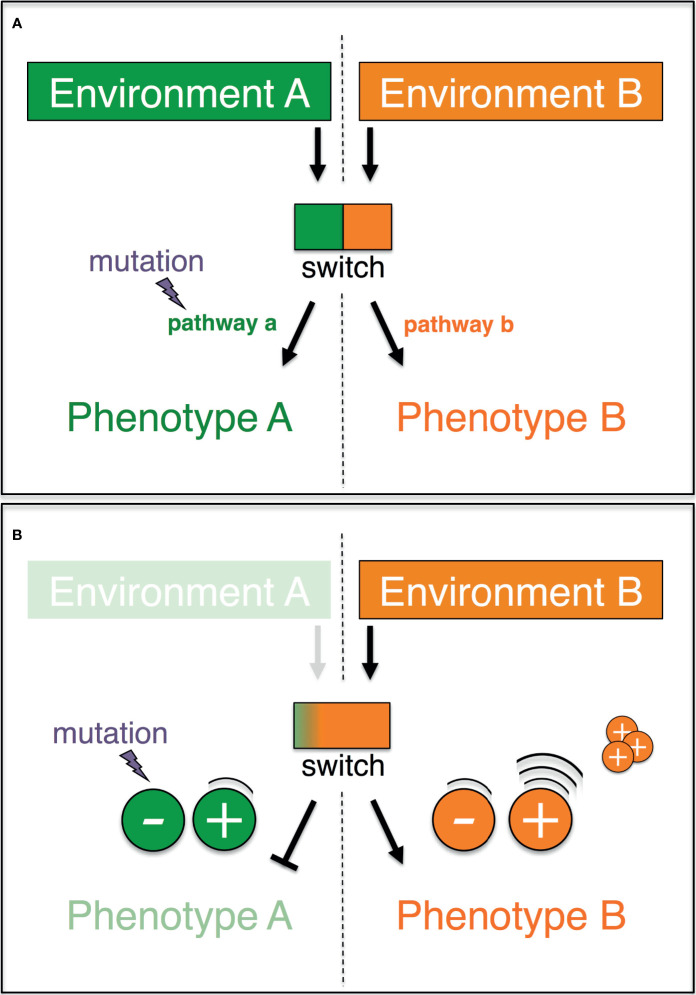
Graphic representation of the plasticity-relaxation-mutation model (Hughes, 2012) and its expanded version (this study). **(A)** A genetic switch controls the expression of two alternate pathways (a, b) leading, respectively, to two alternate phenotypes (a, b) in response to two different environments (a, b). When environment A is no longer encountered by the organism/cell, there is no longer purifying selection against mutations that eliminate pathway a (modified after Hughes, 2012). **(B)** If phenotype A and phenotype B are antagonistically regulated, then the negative molecular regulators of the disfavored phenotype A are redundant and preferentially accumulate mutations. The positive regulators of the adaptive phenotype B are highly expressed, and their copy number may increase as a result of intracellular processes.

An elegant non-Darwinian and non-Lamarckian mechanism that exhibits all of the foregoing properties has been previously proposed (78), and largely overlooked. Dubbed plasticity-relaxation-mutation, this mechanism builds on the idea that environmentally-induced traits may become genetically determined (or assimilated) (79–83), and that phenotypic plasticity precedes the origin of evolutionary adaptations (84, 85). The rationale of this mechanism is simple (**Figure 1A**). Let us imagine two alternative phenotypes (P_A_ and P_B_), each of which is expressed in one of two different environments (E_A_ and E_B_, respectively). An environmentally regulated genetic switch controls the expression of P_A_ or P_B_. Lastly, P_A_-expressing organisms/cells in E_A_ are assumed to exhibit a higher reproductive success relative to those expressing P_B_ in E_A_ (conversely, P_B_ in E_B_ has a higher fitness than P_A_ in E_B_). In these circumstances, the expression of the phenotype P_A_ in the environment E_A_ shelters the molecular determinants of the alternative phenotype P_B_ from purifying selection. Given a sufficiently long exposure to E_A_, the DNA sequences that underlay the unused phenotype P_B_ may accrue silencing mutations, which can spread by random genetic drift. Thus, habitual exposure to an environment can promote the permanent expression of one phenotype while favoring the loss of the alternative phenotype alongside the inactivation of its molecular basis.

The explanatory power of the plasticity-relaxation-mutation mechanism ( (78) and below) may be further expanded when the genetic switch is explicitly treated as non-binary (*i.e.*, a continuum of states) and the regulation of the phenotypes is unpacked (**Figure 1B**). In an environment (E_A_) where P_A_ is favored and the alternative P_B_ is disfavored/unused (with P_A_ and P_B_ being antagonistically regulated), the positive regulators of P_B_ are downregulated. In these circumstances, the persistent nonuse of P_B_ in E_A_ renders P_B_’s negative regulators redundant and hence vulnerable to inactivating mutations. Thus, the negative regulators of a phenotype that is disfavored/unused in a habitual environment are likely to be the first molecular components to be taken out of action by silencing mutations. On the other hand, the persistent up-regulation of P_A_’s positive regulators in the environment E_A_ may favor their sequence amplification *via* physiological intracellular mechanisms (see below). Hence, the positive regulators of a phenotype that is favored in a habitual environment are the most likely to accrue copy number variants. Finally, the molecular inactivation of a disfavored/unused phenotype could have surprisingly wide phenotypic effects. Besides being viewed as individual traits, the alternative phenotypes, P_A_ and P_B_, may each be viewed also as a collection of traits, which were molecularly linked over evolutionary time because of the selective advantage conferred by their functional integration. If so, mutations that inactivate even one or a few components of the molecular basis of, say, P_B_ in E_A_ can at once impact the expression of multiple linked traits.

In sum, the dynamics proposed by the plasticity-relaxation-mutation mechanism support a *use-it* or *lose-it* model where genes/pathways that are persistently not used in an environment culminate in being permanently silenced and possibly physically lost (**Table 1**). In contrast, the genes that are activated in a habitual environment and underlie the favored (adaptive) phenotype can experience amplification, which could be seen as a *use-it* and *improve-it* dynamic. In this model, relaxed purifying selection together with random genetic drift and effectively neutral or nearly neutral mutations play a central role in the adaptive reconfiguration of genomes and phenotypic (individual or integrated) traits.

**Table 1:** Putative mechanisms responsible for the origin of evolved adaptations.

Theory of evolution	Mechanism of adaptation	Driving factor	Predictions (sample)	Refs
***Neo-Darwinism***	Natural selection acts on heritable variability that originates through accidental changes in the genetic material.	Positive Selection, Mutation	Positive selection is necessary for evolutionary adaptation. DNA sequence contributes more to adaptive evolution than epigenetic variants. Mutational trajectories are not influenced by the environment. Adaptive phenotypes are usually built up by a series of relatively small changes. The frequency of potentially advantageous genetic mutations is extremely low. Adaptation is limited by mutations.	(37, 64, 86, 87)
***Neo-Lamarckism***	A new environment directly induces adaptive and heritable phenotypic changes. Environmental epigenetics and epigenetic transgenerational inheritance provide molecular mechanisms for this process.	Environment, Epiallelic change (Mutation)	Selection is not involved in the spread of heritable and adaptive changes that a particular environmental treatment tends systematically to induce. Induced epigenetic changes can be stably transmitted over many generations in the absence of the treatment. Stable epiallelic variants without associated DNA sequence variants are abundant among spontaneous mutations.	(37, 88, 89)
***Use-it or Lose-it***	In an environment where a phenotype is permanently expressed, the molecular basis of alternative phenotypes is relaxed. Mutations that permanently eliminate pathways leading to alternative phenotypes can be fixed by genetic drift. Genes that underlie the favoured phenotype may undergo recombination-mediated amplification.	Environment, Phenotypic plasticity, Purifying selection, Mutation, Genetic drift, Recombination	Positive selection is not necessary for evolutionary adaptation. Phenotypic plasticity precedes the fixation of evolved adaptations. Copy number variants are a frequent contributor to adaptation. Evolved adaptations originate from pre-existing traits that are co-opted for a new function. Loss-of-function mutations are associated with the evolution of phenotypic novelties. Evolutionary adaptation can be achieved even when effective population size is small. Epigenetic silencing of genes involved in the disfavoured pathway could accelerate evolution because it shelters genes from purifying selection.	(87) This study

## The *Use-It* or *Lose-It* Model of Adaptive Evolution Has Considerable Explanatory Power and Makes Testable Predictions

The foregoing propositions are compatible with many observations. For example, they are consistent with empirical findings showing that environment-induced plasticity can promote adaptive evolution (90, 91), that expression variability among environments can affect gene evolution (92), and that a trait’s variance may be controlled by genes that are not directly involved in the trait being considered [the Omnigenic Model (93)]. They align with the increased rate of tandem duplications frequently associated with up-regulated stress-responsive genes in several organisms (94–96) and with the predictable and frequent formation of *de novo* copy number variation in independent experimental evolution lines of yeast (97–99). One fulfilled prediction of the *use-it* or *lose-it* model is that the rate of adaptive molecular evolution scales negatively with the intensity of natural purifying selection (100). Another fulfilled prediction is that an adaptation to a certain environment may limit evolutionary potential under environmental change (101) and can be deleterious in other environments (102–104).

The *use-it* or *lose-it* model accounts for — without depending on — the role of epigenetic changes in favoring the onset of evolved adaptations (78). Epigenetic mechanisms, such as DNA methylation or small RNA-mediated epigenetic modifications, can help directly regulate the proposed coordinated antagonistic expression of P_A_ and P_B_ (105, 106) (**Figure 1**) and may be inherited (107). It can also account for the adaptive contribution of spatial and temporal non-genetic heterogeneity in populations of genetically narrow or uniform cells (28, 108, 109). For example, prolonged proximity to a new and confined source of stress (*e.g.*, inflammation) is expected to locally promote non-genetic changes that are more stable and thus more likely to reoccur across generations compared to changes that occur further apart from the source of stress. Under these circumstances, the *use-it* or *lose-it* model predicts that mutations that silence the molecular basis of the unused phenotype are most likely to accrue locally around, more than further apart from, a changed environment. More broadly, the model makes the testable prediction that non-genetic heterogeneity, the likelihood of genetic inactivation and the loss of phenotypic plasticity all correlate with the intensity/duration of, and distance from, a localized source of stress.

Exposure to a new environment triggers a physiological adaptive response (also known as acclimatization), which in the *use-it* or *lose-it* model shapes evolutionary adaptive trajectories. This means that evolutionary adaptive trajectories could be to some degree predicted *via* ecological studies, *e.g.*, *via* the study of transcriptional variation in response to environmental change, in line with previous suggestions (110). Finally, the *use-it* or *lose-it* model aligns with the widespread evidence for convergence by parallel evolution (111–114). It predicts that individuals/cells that are habitually exposed to the same biotic or abiotic environment (*e.g.*, diet, medication, hygiene levels, pollutants, oxygen concentration, population density) accumulate inactivating mutations in the molecular basis of the same alternative phenotype that is disfavored/unused in that environment. This prediction matches the increasingly acknowledged role of gene loss for evolutionary adaptation (115–122). It also provides a plausible explanation for recurrent mutations in cancer cells (see below), recurring intratumoral phenotypic clusters (77, 123, 124), and the convergence towards the relatively few hallmarks of cancer (125). Importantly, most if not all the evidence presented above is commonly interpreted as, and may indeed be, the result of positive selection. However, the *use-it* or *lose-it* model offers an additional and equally viable interpretation for these observations.

## Physiological Adaptation to the Pre-Tumor Environment

How far can the *use-it* or *lose-it* model take us with regard to improving current understanding of cancer initiation and evolution? Below, we provide a broad-brush overview of events that may unfold during tumorigenesis. This work of synthesis illustrates how the *use-it* or *lose-it* model can be used as a lens to interpret common observations and, more generally, as a valuable guide for gaining insights into cancer adaptive dynamics.

Although the exact timing and order of events that determine tumor initiation is not yet fully elucidated (126), it seems clear that tumorigenesis is a multi-stage (127) cumulative (128) process, and that the onset of cancer depends heavily on the surrounding microenvironment. Indeed, chronic inflammation and other types of long-lasting microenvironmental stresses are strongly associated with an increased risk of cancer (129–131). In healthy somatic cells, microenvironmental stress is expected to induce a stress response. As a part of this response, human cells physiologically upregulate the expression levels of genes such as those encoding HSP70 and p53. The former is a family of proteins, amongst the most conserved across the tree of life (132, 133). The latter is a metazoan invention (134), and its encoding gene is among the most frequently mutated across cancer types in human (135–137). Stress-related proteins such as HSP70 and p53 can play multiple functions in the cell (pleiotropy). For example, HSP70 is also a positive regulator of mitotic cell division (138), whereas p53 negatively regulates the cell cycle (139). Moreover, these functions may be mutually exclusive (antagonistic pleiotropy). In accordance with the above example, stress resistance and cell growth are inversely regulated across the tree of life (110, 140). Finally, stress resistance and cell growth are not isolated biological processes. Energy stores mobilization and cell motility, for example, are evolutionarily and molecularly linked with the cell stress response (141, 142). Instead, processes such as microenvironment sensing, adhesion signaling, programmed cell death, and circadian clock are intimately connected to cell cycle progression (143–145). Given these connections, when the molecular basis of one of these processes is altered, then other interlinked processes may also be affected.

This brief account exemplifies how upon exposure to pre-malignant microenvironmental stress, multipurpose proteins such as HSP70 could take on the role of environmentally controlled genetic switch that is hypothesized in the plasticity-relaxation-mutation mechanism (78) (see above and **Figure 1**). The switch could simply reflect a biased allocation of pleiotropic factors between the competing demands of growth and stress resistance/somatic maintenance *e.g.*, following post-translational modifications (146, 147). In any case, proteins with critical roles in stress response and cell cycle progression such as HSP70 may fail to accurately mediate cell division in a stressful environment (148). This is expected to hinder cell growth and may generate ploidy alterations (149) and copy number variation (98) in a context, the pre-malignant environment, where stress resistance is the adaptive phenotype. According to the *use-it* or *lose-it* model, the positive regulators of this stress-resistance phenotype are upregulated and may accrue structural changes (**Figure 1B**). Instead, cell growth is the alternative disfavored/unused phenotype, whose expression is antagonistically regulated *via* epigenetic changes, and whose molecular negative regulators preferentially accrue silencing DNA mutations (**Figure 1B**).

The above dynamics align well with observations from long-term experimental evolution studies. In one example, independently evolving yeast populations accumulate adaptive copy number variants in response to stress. These variants predictably emerge across replicates and appear to result from DNA replication-mediated processes (97). These findings and others (150–153) align with the suggestion that in a constant environment active transcription can contribute to the formation of copy number variants (**Figure 1B**). In a constant environment, these variants can be repeatedly generated across individuals/cells and hence they can increase frequency in a population even when the power of selection is weaker relative to the power of genetic drift.

In a second example, independently evolved yeast clones frequently undergo adaptive self-diploidization in response to a chronically stressful environment (*i.e.*, limiting glucose) (154). Self-diploidization is strongly associated with enhanced stress resistance (155) and produces similar phenotypic effects as those observed when cell cycle progression-related genes are repressed (156). These obervations hint at the expected link between stress response and cell growth in yeast. Furthermore, independently evolved yeast clones with no self-diploidization were reported to accumulate a large number of large-effect adaptive mutations in a few genes that affect cell growth (154). Not only do these recurrent mutations inactivate preferentially negative regulators of the nutrient-responsive Ras/PKA pathway, but mutations that decrease the activity of Ras/PKA genes are known to have strong pleiotropic effects and enhance yeast’s response to stress (157–160). These dynamics align with those described in **Figure 1B**. Under the ND model, the discussed mutations accrue as a result of positive selection. Under the *use-it* or *lose-it* model, relaxed negative selection favors the accrual of these growth-inhibiting mutations, which in a stressful environment stabilize the enhanced stress response.

In a third and last example, a constant environment with predictable nutrient supply is reproducibly associated with perturbed/disrupted environment-sensitive and growth rate-governing signaling pathways in the single-celled ciliate *Paramecium* (161) and yeast (162). In the yeast study, clones adapted to the constant environment show also reduced viability in a fluctuating environment where nutrient abundance varies (162). These findings align with the *use-it* or *lose-it* model: habitual exposure to a constant environment is expected to wear down the ability to respond to environmental changes. This latter aspect may play a crucial role in oncogenic transformation (see below).

## Mechanisms Underlying Oncogenic Transformation

But how can the foregoing physiological adaptive dynamics lead to an oncogenic transformation? The *use-it* or *lose-it* model generates a specific prediction based on a few previous observations. First, it is widely known that the growth-inhibitory activity of p53 is elevated in the presence of stress but drops during tumorigenesis (163). Second and last, across multiple cancer types the p53-encoding gene *TP53* preferentially accrues inactivating mutations, many at the early phases of tumorigenesis (137, 164). Based on this and the above-presented work of synthesis, the *use-it* or *lose-it* model predicts that the emergence of tumors is coupled with — or even prompted by — a “*stress resistance-cell growth axis*” that tilts in favor of cell growth. Because stress resistance is no longer the favored/used phenotype in the newly formed tumor microenvironment (more details below), its molecular basis becomes the preferential target of inactivating mutations (**Figure 1**).

But what would prompt the putative tilt in the stress resistance-cell growth axis? Although only targeted experiments can reveal the precise causes, the *use-it* or *lose-it* model offers a simple explanation, which can guide future investigations. Under this model, sustained repression of cell growth in the stressful pre-tumor microenvironment favors the accumulation of cancer-associated inactivating mutations in genes that encode cell-growth negative regulators such as p53 (**Figure 1B**). The duration of exposure to the pre-tumor microenvironment is crucial for inactivating mutations to accrue. Thus, the *use-it* or *lose-it* model predicts that tumors irreversibly emerge when the level of environmental stress remains elevated for a sufficiently long time — a time span that might even extend for years (137). The same rationale can be readily used to explain adaptation at later phases of cancer development, *i.e.*, developed tumors would be most likely to evolve genetically-determined adaptations when their microenvironment remains constant for a sufficiently extended time (165). This implies that long-term exposure to constant drug therapies may be a shortsighted anti-cancer approach, in line with current views (166) and emerging therapies (167).

Why should stress resistance be dampened in the emerging tumor microenvironment? Because there are no indications, to our knowledge, that the pre-tumor environmental stress disappears during tumorigenesis, the hypothesized tilt in the stress resistance-cell growth axis can only indicate that newly emerged tumor cells have evolved a considerable level of *insensitivity* to stress (which would effectively dampen or turns off stress response). This may reflect a dampened sensitivity to extracellular and intracellular information and/or a partial loss of contact with the microenvironment. Several observations are in line with this inference. For example, dysregulated signaling is widely recognized as an oncogenic mechanism (125), with expected effects on the cell’s metabolic and epigenetic circuits (168). Also, the loss of polyploidy can flag a reduced sensitivity to stress (*e.g.*, DNA damage). Polyploidy enhances a cell’s ability to survive in stressful conditions (169) and is frequently observed in pre-malignant lesions (170). However, polyploidy is frequently lost during the early steps of tumorigenesis (171) *via* chromosome missegregation (172), expecially in cells where the function of p53 is down-regulated or absent (173). Furthermore, the stress-inducing pre-tumor microenvironment can activate the epithelial-to-mesenchymal transition program [reversible when the inducing stress is removed (174)] which mediates, *inter alia*, the loss of epithelial characteristics (*e.g.*, cell-cell adhesion) (35, 175). The hypothesized reduced sensitivity to the microenvironment during oncogenic transformation is further consistent with evidence supporting a model of cancer driven by tissue disruption (176), as well as with the proposed link between the age-related destabilization of the multicellular organizational architecture and cancer (177, 178). It is also consistent with the suggestion that the onset of cancer reflects a transition to unicellularity (179–181). More specifically, it has been suggested that a healthy somatic cell within a tissue is *non-evolving* unless this cell breaks loose and thus acquires a self-defined fitness (72). The hypothesized reduced cell sensitivity to biological information may favor the acquisition of this self-defined fitness alongside the activation of molecular programs that date back to unicellular ancestors (182–184).

What mediates enhanced cell proliferation in the emerging tumor microenvironment? As stated above, the inactivated or inhibited p53 no longer carries out its anti-proliferative transcriptional program. This is expected to promote cell growth, in addition to influencing a plethora of other cellular processes (**Box 2**). Additionally, putative genetic switches such as HSP70, which is typically highly expressed in tumors (133), may be re-allocated from chiefly enhancing stress response in the pre-tumor environment to primarily promoting mitotic division in the tumor microenvironment. In line with this, HSP70 plays a key role in cancer initiation and progression (185). Moreover, its loss prevents malignant transformation (185). More in general, environmentally-controlled reallocations along the stress resistance-cell growth axis are consistent with two highly correlated phenotypes which are detected in cancer and unicellular systems such as the ciliate *Paramecium* (148): dormancy and elevated stress resistance *vs*. rapid proliferation and reduced stress resistance.

Box 2 **Bridging p53 inactivation, cancer and ontogenesis with the *use-it* or *lose-it* model.**

Several observations indicate that the functional inactivation of p53 can rewire cells. For example, p53 inactivation/inhibition disfavors DNA repair and apoptosis (186), may favor cell migration and invasion (187, 188), induces the de-repression of transposable elements (189–191), and facilitates the survival and accumulation of de-polyploidized/aneuploid cells (172, 173, 192, 193) and immune evasion (194, 195). This massive cascade of events may reflect genome instability in somatic cells that, because of the inoperative p53, are reprogrammed to generate induced pluripotent stem cells (196). In a seemingly alternative fashion, these events may also reflect the activation of an evolutionary conserved program of unicellular survival against unfavorable changes (197).

Can the foregoing distinct views be linked under a unifying perspective? This is probably only possible when the hypothesized program of unicellular survival against unfavorable changes matches the transcriptional program of embryonic stem cells. Indeed, the transcriptional program of tumor cells does overlap with that of embryonic stem cells (198, 199). Similar to cancer cells, early embryo cells exhibit a reduced expression of p53 and a similar activation of LINE-1 elements (200). Moreover, placental cells exhibit several cancer-like features, *i.e.*, genome instability (201), invasiveness and suppression of immune responses (202, 203), methylome and vascular remodeling (204, 205). Further, tumor cell reprogramming to pluripotency — an event that is observed in different phases of cancer biology — most likely enhances cell resistance [*e.g.*, to therapy-induced stress (206)]. In sum, it is plausible that the functional inactivation of p53 in somatic cells triggers some non-random dynamics that reflect a throwback to the early stage of embryogenesis.

Leveraging these similarities between tumor cells and early embryo/placental cells, the *use-it* or *lose-it* model successfully predicts that early embryo cells are habitually exposed to an environment that closely resembles a tumor microenvironment [*e.g.*, hypoxia (207, 208)]. Despite the many similarities between tumor cells and early embryo/placental cells, tumorigenesis typically does not take place during fetal/placental development. This suggests that the process of embryonic development unfolds in the presence (absence) of factors that prevent (promote) tumor initiation (209, 210). Based on the rationale of the *use-it* or *lose-it* model, differences in the properties of the microenvironmental stress during embryogenesis and tumorigenesis (*e.g.*, intensity, persistence) may crucially contribute to either building a multicellular system or promoting cancer (211). If so, then we anticipate that a better understanding of the mechanisms driving cancer evolution can help gain insights into the mechanisms governing embryo development and vice versa.

## Implications for Translational Application

Understanding whether and when cancers adapt *via* a neo-Darwinian model, or a different model of adaptive evolution can have serious practical consequences for patients. At present, recurring mutations that inactivate specific genes in a patient’s cancer cells are assumed to flag positive selection-driven adaptations, say an evolved capacity to escape the immune system. Based on this common assumption, inactivated genes are labeled as tumor suppressors and oncologists may adjust or change altogether the cancer treatment to which the patient is subjected. For example, a patient may be treated with different chemotherapeutical drugs or a cocktail thereof to circumvent the hypothetical evolved capacity to evade immune response.

But what if the underlying assumption rooted in the neo-Darwinian paradigm is contentious or, worst, inaccurate? The following example illustrates this possibility. A recent study found that inactivating mutations in metastatic/relapsed breast cancer cells preferentially target the genes *JAK2* and *STAT3*, and more in general, the JAK-STAT signaling pathway (212). Following the current *modus operandi*, Yates et al. labeled *JAK2* and *STAT3* as tumor suppressor genes in metastatic/relapsed breast cancer. Further, as the loss of *JAK2* was previously shown to lead to a total loss of functional response to interferon gamma (213), Yates et al. proposed — reasonably, under an underlying neo-Darwinian paradigm — that the recurring inactivating mutations in the JAK-STAT signaling pathway contribute to disease progression by allowing cancer cells to adaptively escape host immunity. The *use-it* or *lose-it* model offers a different, yet equally viable interpretation for these same findings. If cells with nonfunctional *JAK2* are unable to respond to interferon gamma as previously suggested (213), then the preferential inactivation of *JAK2* reported by Yates *et al.* can reflect an interferon gamma-poor environment. In other words, rather than being positively selected to escape the immune system (neo-Darwinian model), the recurrent inactivation of JAK-STAT signaling in metastatic/relapsed breast cancer may flag a weakened immune system and favor survival in the interferon-gamma-poor environment (*use-it* or *lose-it* model).

This alternative interpretation fits well with the negative impact of cancer and chemotherapy on the immune system (214). It also cautiously suggests an approach to counter this adaptive cancer phenotype. Treating the patient with interferon gamma could encourage metastatic breast cancer cells to re-activate a silenced or dampened JAK-STAT signaling, while at the same time mitigating the overexpression of the alternative (adaptive) phenotype (215, 216). It is worth noting that the antitumor effects of an increased interferon gamma approach have been already verified. Treating breast (and other) cancer patients with interferon gamma can help sensitize cancer cells to apoptosis, facilitating their elimination with additional drugs (217–221). Moreover, the molecular basis of the alternative (adaptive) phenotype may encompass the PI3K/AKT/mTOR signaling pathway. The latter inference is drawn from some observations: the increased activity of the PI3K/AKT/mTOR signaling pathway is frequently observed in breast cancer patients (222), it increases tumor resistance to multiple drugs (223, 224), and PI3K/mTOR inhibitors activate the JAK/STAT signaling pathway (225).

## Conclusions

Cancer is an evolutionary adaptation of malignant cells to their microenvironment. Thus, an adequate understanding of the mechanisms that mediate adaptive biological responses is indispensable to make effective progress against cancer. Inspired by a wealth of empirical and theoretical cancer and evolutionary studies, here we build on a non-Darwinian and non-Lamarckian model of adaptive evolution (78) to gain insights into therapeutic resistance and other steps of cancer biology.

In the neo-Darwinian model, ecology and evolution are treated separately (226), chance plays a central role in the emergence of mutations, and environmental adaptation is achieved *via* positive selection of spontaneous mutations (37). In the revived plasticity-relaxation-mutation model (78) — here expanded and renamed with a more intuitive *use-it* or *lose-it* — physiological and evolutionary changes are integrated, mutations preferentially accrue in those genomic loci that upon environmental change experience a change of selective regime (*i.e.*, relaxed purifying selection), and adaptation is achieved by losing the phenotype that is unused in a habitual environment.

The *use-it* or *lose-it* model has a considerable explanatory power. Its theoretical propositions align, *inter alia*, with the idea that that enduring environmental conditions can affect simultaneously health and evolutionary trajectories (227). They can explain the parallel evolution of similar traits in response to the same environment [*e.g.*, recurrent losses in response to the same anti-cancer therapy (228)], are compatible with the observation that non-genetic heterogeneity can promote transient and rapid adaptation to environmental changes (229, 230), and with evolution-based therapeutic strategies such as the “ersatzdroge” strategy to counter drug-resistant phenotypes (231). In this latter strategy, cells with an evolved adaptation to a certain environment (*e.g.*, resistance to a toxic drug) are outcompeted by the sensitive cells when exposed to a non-toxic version of the same drug (a new environment). The *use-it* or *lose-it* model can help generate a conceivable and coherent mechanistic account of tumorigenesis, and when compared to the neo-Darwinian model it can produce different interpretations of the same empirical observations, thereby encouraging different clinical interventions. These differences deserve special attention given the increasingly widespread use of personalized oncology/medicine and its role in informing clinical treatment.

Finally, the *use-it* or *lose-it* model suggests that the conversion of a healthy cell into a cancerous cell is neither abrupt nor accidental. Rather, it is the result of an environmentally induced process that is for one traceable and for another reversible. If this model is correct, then tracing epimutations induced by a habitual exposure to microenvironmental stress should represent a powerful strategy to anticipate the emergence of cancer. Additionally, in a world where obesity is the first cause of cancer (232, 233), changes of inflammation-promoting lifestyle and dietary regimes would help reverse and reduce the induction of cancer-related mutational and adaptive events (234). Twenty-five years ago Lucien Israel wrote: “*Killing the last cancer cell without killing the host is an objective that has not yet been reached*” (197). In large part, this is still true today (235). Considering the foregoing propositions, we urge a profound reconceptualization of anti-cancer therapies. Instead of aiming to kill cancer cells, we suggest that anti-cancer therapies should aim to prevent or alter the (micro)environmental conditions that spawn and/or help preserve cancer cells. In this context, the characterization of genetic and non-genetic cancer variation would no longer serve to design toxic drugs or debilitating treatments tailored to specific molecular targets. Rather, it would help infer the key properties and the vulnerabilities of the tumor-supporting environment, which non-toxic means could help modify.

## Data Availability Statement

The original contributions presented in the study are included in the article/supplementary material. Further inquiries can be directed to the corresponding author.

## Author Contributions

FC conceived of the presented idea and developed the theory. All authors contributed to the article and approved the submitted version.

## Funding

FT is supported by the MAVA Foundation and an ANR TRANSCAN (ANR-18-CE35-0009).

## Conflict of Interest

The authors declare that the research was conducted in the absence of any commercial or financial relationships that could be construed as a potential conflict of interest.

## Publisher’s Note

All claims expressed in this article are solely those of the authors and do not necessarily represent those of their affiliated organizations, or those of the publisher, the editors and the reviewers. Any product that may be evaluated in this article, or claim that may be made by its manufacturer, is not guaranteed or endorsed by the publisher.
